# How Does Diagnostic Accuracy Evolve with Increased Breast MRI Experience?

**DOI:** 10.3390/tomography9060162

**Published:** 2023-11-06

**Authors:** Tong Wu, Afsaneh Alikhassi, Belinda Curpen

**Affiliations:** Breast Imaging Division, Medical Imaging Department, Sunnybrook Health Sciences Centre, University of Toronto, Toronto, ON M4N 3M5, Canada; tong.wu@vch.ca (T.W.); belinda.curpen@sunnybrook.ca (B.C.)

**Keywords:** MRI, PPV3, experience, diagnostic accuracy, BI-RADS 4, high-risk lesion, breast cancer

## Abstract

**Introduction:** Our institution is part of a provincial program providing annual breast MRI screenings to high-risk women. We assessed how MRI experience, background parenchymal enhancement (BPE), and the amount of fibroglandular tissue (FGT) affect the biopsy-proven predictive value (PPV3) and accuracy for detecting suspicious MRI findings. **Methods:** From all high-risk screening breast MRIs conducted between 1 July 2011 and 30 June 2020, we reviewed all BI-RADS 4/5 observations with pathological tissue diagnoses. Overall and annual PPV3s were computed. Radiologists with fewer than ten observations were excluded from performance analyses. PPV3s were computed for each radiologist. We assessed how MRI experience, BPE, and FGT impacted diagnostic accuracy using logistic regression analyses, defining positive cases as malignancies alone (definition A) or malignant or high-risk lesions (definition B). **Findings:** There were 536 BI-RADS 4/5 observations with tissue diagnoses, including 77 malignant and 51 high-risk lesions. A total of 516 observations were included in the radiologist performance analyses. The average radiologist’s PPV3 was 16 ± 6% (definition A) and 25 ± 8% (definition B). MRI experience in years correlated significantly with positive cases (definition B, OR = 1.05, *p* = 0.03), independent of BPE or FGT. Diagnostic accuracy improved exponentially with increased MRI experience (definition B, OR of 1.27 and 1.61 for 5 and 10 years, respectively, *p* = 0.03 for both). Lower levels of BPE significantly correlated with increased odds of findings being malignant, independent of FGT and MRI experience. **Summary:** More extensive MRI reading experience improves radiologists’ diagnostic accuracy for high-risk or malignant lesions, even in MRI studies with increased BPE.

## 1. Introduction

Breast cancer is the most prevalent cancer diagnosed in women, accounting for 25% of all new cancer cases diagnosed in women in Canada in 2022 [1]. BRCA1 and BRCA2 mutation carriers represent 5–10% of females with breast cancer. By the age of 80, BRCA1/2 mutation carriers have a 70% risk of developing breast cancer, which is seven times higher than the general population (10%) [2]. Additionally, one in three individuals with BRCA1/2 mutations will develop breast cancer by the age of 50 [2]. Other genetic variations, such as Li–Fraumeni, Cowden, Peutz–Jeghers, diffuse gastric, and lobular breast cancer syndromes, are also associated with increased risks of breast cancer [3].

In July 2011, our province established a screening program specifically designed for high-risk women, offering annual digital mammography and breast MRIs to women between the ages of 30 and 69. Eligible individuals include those who are known carriers of mutations such as BRCA1 and BRCA2, TP53, PTEN, and CDH1, those who have first-degree relatives with these mutations and have opted not to undergo genetic testing, those who have undergone risk assessment by a genetic clinic using the IBIS model or BODICEA and have a personal lifetime risk of breast cancer of 25% or higher, or those who have received radiotherapy to the chest before the age of 30 at least 8 years prior [4]. The program also extends to women with syndromes such as Lynch and Li–Fraumeni. Our institution is currently 1 of the 28 sites in our province that provides access to this screening program.

Although breast MRI exhibits higher sensitivity in detecting breast cancer than other imaging modalities such as mammography and ultrasound, it is also associated with increased recall rates and biopsies of benign lesions, with a wide BI-RADS benchmark PPV3 range between 25 and 50% [5], and a published local provincial PPV3 of 16.9% from 2011 to 2020 [6]. This study aims to determine the positive predictive value (PPV3) of screening breast MRI conducted by radiologists at our center for women at higher risk. There are limited studies that evaluated the effect of radiologist experience on diagnostic accuracy [7,8]. Therefore, we also aim to evaluate how diagnostic accuracy in identifying suspicious lesions evolves with increased MRI reading experience. Studies have shown that higher background parenchymal enhancement (BPE) is associated with lower specificity [9] and that an increased amount of fibroglandular tissue (FGT) may decrease accuracy [10,11]. Therefore, we also aim to assess the impact of BPE and FGT on the radiologists’ ability to accurately detect cancers or high-risk lesions in screening breast MRIs.

## 2. Methods

REB approval was obtained from the Sunnybrook Health Science Centre Research Ethics Board, and the need for consent was waived.

**Figure 1:**
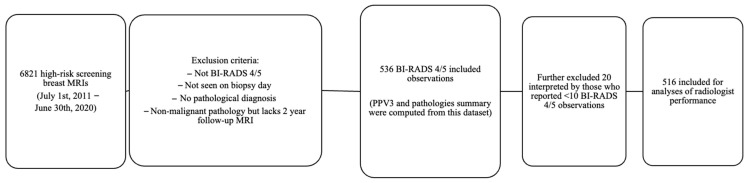
Flowchart of included and excluded observations for data analysis.

A retrospective cross-sectional study was conducted involving all high-risk screening contrast-enhanced MRIs performed at our institution from 1 July 2011 to 30 June 2020, with a BI-RADS 4 (indicating suspicion for malignancy) or BI-RADS 5 (highly suggestive of malignancy) classification in the MRI report. A chart review was conducted for these cases. Histopathology results obtained within 12 months from fine-needle aspiration, core biopsy, excisional biopsy, or prophylactic mastectomies were retrieved from our institution’s electronic health record system. Cases without available pathology results, including lesions that were no longer visible on the day of the biopsy, were excluded. The pathology results were categorized into three main groups: malignant, high-risk, and benign. Malignant cases included invasive carcinoma, ductal carcinoma in situ (DCIS), and lymphoproliferative disease. The high-risk group comprised lobular carcinoma in situ (LCIS), atypical lobular hyperplasia (ALH), atypical ductal hyperplasia (ADH), papillary lesions with or without atypia, radial scar, and flat epithelial atypia (FEA). Benign and high-risk lesions that did not undergo surgical excision but demonstrated stability on follow-up MRIs over a period of 2 years were considered truly benign. Cases were excluded if there was no surgical pathology available or if there were less than 2 years of follow-up MRI data to confirm stability. Observations of BI-RADS 4/5 detected during diagnostic/follow-up MRIs of the high-risk population were not included in this study, as shown in Figure 1.

### 2.1. Imaging Technique

From 2011, all MRI examinations were performed on a 1.5T MRI scanner (Signa Excite, GE HealthCare Medical Systems, Covington, GA, USA) with a standard bilateral dedicated breast coil. The sequences included precontract sagittal T1, T2-weighted images with fat suppression, and dynamic contrast-enhanced (DCE) T1-weighted imaging sequences. An amount of 0.1 mmol/kg IV gadolinium contrast was administered as a bolus, and four post-contrast scans, MIP, and 3D reformatted images were obtained. Since 2018, we have changed to 1.5T MRI (Siemens Healthineers, Erlangen, Germany) with the Identical sequences.

### 2.2. Chart Review

The following variables were extracted from each MRI report: the interpreting radiologist, BPE classified as minimal, mild, moderate, or marked, and the amount of FGT categorized as almost entirely fat (A), scattered (B), heterogeneously dense (C), or extremely dense (D) FGT. In cases where the BPE level or the amount of FGT was missing from the MRI reports, a retrospective assignment was made by a staff breast radiologist. If the BPE level was described as falling between two levels, the higher of the two levels was recorded. In instances where multiple findings were classified as BI-RADS 4/5 within the same MRI report, each finding was treated as a separate observation in the dataset, maintaining consistency in terms of the radiologist, patient profile, BPE, and amount of FGT.

### 2.3. Data Analysis

The total number of high-risk screening breast MRIs was calculated and recorded for each calendar year. The overall positive predictive value (PPV3) and PPV3 stratified by the year of the BI-RADS 4/5 observations that underwent biopsy were calculated. The total count of MRI BI-RADS 4/5 observations was compiled for each radiologist.

The MRI experience of the radiologist on the day of interpretation is determined by calculating the difference in years (obtained by dividing the difference in days by 365) between the examination date and the date when they initially began reporting breast MRIs as a staff member.

We employed two different definitions to classify positive cases. In definition A, positive cases consisted of BI-RADS 4/5 observations resulting in malignant pathologies. In definition B, positive cases included BI-RADS 4/5 observations resulting in either malignant or high-risk pathologies. Using these definitions, we conducted logistic regression analyses, both with single and multiple variables, to investigate potential correlations between the radiologists’ MRI experience (measured in three increments: 1 year, 5 years, and 10 years), BPE, and the amount of FGT with diagnostic accuracy. Radiologists who reported fewer than ten observations were excluded from these logistic regression analyses.

Descriptive analyses, graphs, and regression analyses were conducted using IBM SPSS Statistics Version 29 and SAS^®^ OnDemand for Academics. Statistical significance was defined as *p*-values less than 0.05.

## 3. Results

### 3.1. PPV3

Out of the 6821 high-risk screening breast MRIs performed at our center between 1 July 2011 and 30 June 2020, a total of 536 BI-RADS 4/5 observations with pathological tissue diagnosis were recorded. The number of high-risk screening MRIs and PPV3 varied by exam year during the period from 1 July 2011 to 30 June 2022, with the lowest PPV3 rate of 8% occurring in 2018 and the highest rate of 43% in 2020 (Table 1).

### 3.2. Pathology Summary

A total of 77 observations (14.4% of 536) resulted in malignant pathologies, comprising 31.2% (24/77) DCIS, 67.5% (52/77) invasive breast cancer, and 1.3% (1/77) lymphoproliferative disease. In contrast, a total of 459 observations (85.6% of 536) revealed non-malignant pathologies. Among the non-malignant pathologies, 51 cases (9.5% of 536) were identified as high-risk lesions, including 14 cases of ADH, 10 cases of ALH, 13 cases of papillary lesions, 2 cases of FEA, 1 case of LCIS, 9 cases of radial scars, 1 case with both ALH and a papillary lesion, and 1 case with ALH and FEA.

### 3.3. Individual Radiologist Observations

These observations were made by a group of 11 radiologists, with the number of observations ranging from a minimum of one to a maximum of 133. The average number of observations per radiologist was 49, with a standard deviation of 49 (Table 2). 

### 3.4. Radiologist MRI Experience and Performance

**Figure 2:**
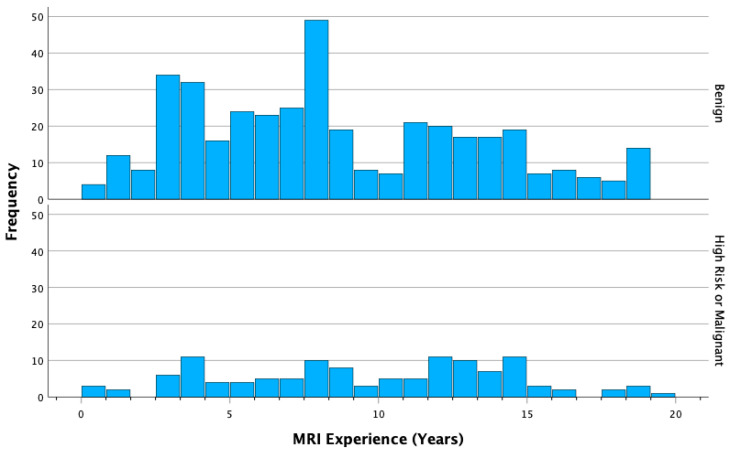
Distribution of MRI experience (years) among correct (high-risk and malignant) and incorrect (benign) cases.

For the analysis of radiologist MRI experience and performance, a total of 20 observations made by five radiologists who reported fewer than ten observations were excluded, resulting in a final sample size of 516 observations (N = 516). Individual PPV3 values for the radiologists ranged from 6% to 22%, with an average of 16% and a standard deviation of 6% (Table 2). As demonstrated in Table 3 and Table 4 when considering only malignant lesions as positive cases (definition A), there was no significant correlation found between positive cases and MRI experience. However, when both malignant and high-risk pathologies were considered positive cases (definition B), a significant correlation was observed between positive cases and increased MRI experience. For each additional year of MRI experience, the odds ratio (OR) was 1.05. Furthermore, there was an OR of 1.27 for a 5-year increase in MRI experience and an OR of 1.61 for a 10-year increase in MRI experience (*p* = 0.03 for all). Multiple variable regression analysis from Table 5 confirmed the independence of this correlation from the effects of BPE, with an OR of 1.05 (*p* = 0.03). The distribution of MRI experience in years, categorized as correct (high-risk and malignant) versus incorrect (benign) pathologies, is illustrated in Figure 2.

### 3.5. Levels of BPE and Amount of FGT and Diagnostic Accuracy

The frequencies of BPE levels and amount of FGT are presented in Table 6. The moderate level of BPE was the most common, accounting for 45.2% of cases, while the heterogeneously dense amount of FGT was the most prevalent, representing 50.3% of cases.

Compared to BI-RADS 4/5 findings detected in breasts with a marked level of background parenchymal enhancement, the odds of the finding being malignant (definition A) are 5.6 times higher if there is minimal BPE (*p* = 0.004) and 4.2 times higher if there is mild BPE (*p* = 0.01) (Table 4). Multiple variable regression analysis demonstrated that this correlation remains significant and independent of the radiologist’s MRI experience and the amount of FGT (Table 5). There is no significant difference in the diagnostic accuracy between moderate and marked levels of BPE (*p* = 0.30) (Table 4). Similar trends are observed between minimal or mild BPE levels and accuracy when positive cases include both malignant or high-risk lesions (definition B), but these trends do not reach statistical significance (OR 2.4, *p* = 0.08 and OR 2.2, *p* = 0.08, respectively, Table 5).

When considering only malignant lesions as positive cases (definition A), compared to patients with extremely dense FGT, the odds of the finding being malignant increase by a factor of 4.2 (*p* = 0.008) in those with almost entirely fatty breasts, and by a factor of 3.4 (*p* = 0.004) in those with scattered FGT (Table 4). However, these correlations also lose significance in the multiple variable regression model when considering BPE (Table 5). On the other hand, when positive cases include both malignant and high-risk lesions (definition B), a similar trend is observed, indicating increased accuracy with decreased FGT. The odds of the finding being a malignant or high-risk lesion in heterogeneously dense FGT are 2.0 times higher than in those with extremely dense FGT (*p* = 0.02, Table 4). This correlation remains significant in multiple variable regression analyses, independent from BPE (Table 5).

## 4. Discussion

Our center’s overall positive predictive value (PPV3) of 14.4% from July 2011 to June 2022 is lower than the BI-RADS benchmark of 20–50% [5]. However, it is comparable to the published provincial PPV3 of 16.9% (11.8–23.1% CI) from July 2011 to June 2020 [6]. 

Based on 11 years of radiology–pathology correlated data, our retrospective study on high-risk screening contrast-enhanced MRIs demonstrated that increased MRI reading experience enhances the diagnostic accuracy of BI-RADS 4/5 findings. The level of experience of each screening study was computed by the difference between the date of the MRI screening exam and the date the interpretating radiologist started interpretating breast MRIs. We observed a multiplicative effect of time: with 1 year, 5 years, and 10 years of increased MRI experience, the odds of the radiologists’ BI-RADS 4/5 observations yielding high-risk or malignant pathologies increased by 5%, 27%, and 61%, respectively. 

Many studies in the literature investigate the diagnostic accuracy of breast MRI regarding the morphologic and dynamic criteria [12,13,14]. However, limited studies included the influence of reader experience on diagnostic accuracy [7,8]. In a survey by Marino, M.A. and at. al., 4 radiologists and 100 subjects were involved; no high-risk screening MRI was included in that study. The radiologists were classified according to the number of cases read before this study with histological verification. Using the BI-RADS lexicon, the expert reader performed significantly better than all less-experienced readers [7].

Pascal A.T. Baltzer et al. included 259 lesions in 217 patients, with no high-risk screening case and with 6 readers. They classified readers based on the number of breast MRIs reported in a year. Experienced radiologists performed best in mass lesions (versus intermediate experienced: *p* = 0.0288, versus low experienced: *p* = 0.0128). In non-mass lesions, experienced and intermediate readers had similar diagnostic performance (*p* = 0.816), while both groups were superior to the low-experience group (*p* = 0.0124, *p* = 0.007) [8].

We also found that increased BPE hurt the radiologist’s accuracy in classifying BI-RADS 4/5 observations. This finding aligns with a previous study by Ray et al., which demonstrated an association between moderate or marked BPE and higher rates of abnormal interpretations, biopsy procedures, and lower specificity [9]. What sets our study apart is that we further demonstrated that this correlation remains significant regardless of radiologist experience or the amount of FGT. At our institution, high-risk screening MRIs are scheduled during the second week of the menstrual cycle, aiming to minimize the level of BPE. It is worth noting that increased BPE has been extensively investigated as a risk factor for breast cancer [15]. Therefore, efforts to develop more reproducible methods for quantifying BPE could potentially help reduce this variability [16].

Although a large amount of FGT has been shown to harm the radiologist’s accuracy in identifying malignant lesions in mammography, no consistent result was available for MRI [10,11,17]. Our study suggests that this correlation is dependent on the association between the amount of FGT and BPE, as supported by prior literature [18]. A similar trend was observed when positive cases included both malignant and high-risk lesions (definition B). However, the only significant correlation observed was between heterogeneously dense FGT and extremely dense FGT, and this correlation was independent of BPE. We believe that this can be attributed to the fact that variations in BPE do not fully account for the variations in the amount of FGT. It is possible that the correlations between different breast densities and diagnostic accuracy did not reach statistical significance in our study due to the limited sample size.

The cohort in our study exhibited a high false positive rate for BI-RADS 4/5 observations (85.6%), which is consistent with the false positive rate of 83.1% (113/136) reported in the published provincial data from 2011 to 2013 [6]. Nonetheless, it is noteworthy that 9.5% of the BI-RADS 4/5 observations in our cohort yielded high-risk pathologies, and some of these findings would have impacted clinical management based on standard recommendations [19,20]. Our rate of high-risk lesions is slightly lower than a recently published multi-center study conducted in three other hospitals within the same city, which reported a rate of 19.6% (43/219) [21]. However, it is important to note that their study included MRI indications other than high-risk screening, such as staging, surveillance, and problem-solving [21]. In our clinical practice, non-classical LCIS, papillary lesions with atypia or palpable papillary lesions, and ADH are typically surgically excised. Those with ALH undergo closer clinical follow-up. Benign papillary lesions without atypia do not require excision but undergo imaging follow-up [22]. Classic LCIS can either undergo clinical and imaging follow-up or be excised. Traditionally, FEA lesions were excised, but now observation is also a valid option [23].

The high positive predictive value (PPV3) observed in the first half of 2020 could be partly attributed to the outbreak of the COVID-19 pandemic. During this time, individuals with higher risk profiles who were more concerned about their health actively sought screening MRIs, while those with lower risk profiles tended to postpone or delay their screening exams.

### Limitations

The positive association between increased MRI experience and higher odds of BI-RADS 4/5 observations yielding malignant pathologies, although observed in our study, did not reach statistical significance. This lack of significance may be attributed to the small sample size utilized. However, it is worth noting that a significant correlation was found between the odds of BI-RADS 4/5 observations yielding high-risk or malignant lesions collectively. This finding is significant since the presence of high-risk lesions often influences clinical management decisions and can impact patient choices, as indicated by previous studies [21,24]. It is important to acknowledge that the observed correlation between increased MRI experience and improved diagnostic accuracy followed a multiplicative pattern. It is worth mentioning that such a pattern would be expected in any logistic regression analysis with a statistically significant positive OR [25].

Several factors can complicate the assessment of improved diagnostic accuracy in radiologists. Firstly, there may be variation in the training received by radiologists, with more recent graduates potentially benefiting from more structured residency programs compared to those who completed their training decades ago, relying more on on-the-job learning. Additionally, studies have demonstrated that even expert breast radiologists exhibit a significant level of inter-observer variability regarding BI-RADS classification [26]. In our study, the MRI reading experience of the radiologists was calculated as the difference between their starting date and the exam day; however, this oversimplification fails to capture the nuances. As seen in our cohort, the number of breast MRIs a radiologist reads also depends on factors such as part-time or full-time work status and whether they solely focus on breast imaging or practice across other radiology subspecialties.

Furthermore, our data spanning from 2011 to 2022 encompass different stages of various radiologists’ careers. As of July 2011, the breast MRI experience of our radiologists ranged from less than 1 year to 11 years. Additionally, assessing factors such as BPE and the amount of FGT relies on qualitative measures assigned by individual radiologists, introducing further variability into the dataset.

Our analysis focused solely on BI-RADS 4/5 observations, and we did not investigate the association between MRI experience or BPE with BI-RADS 3 observations. A study by Hambly et al. previously examined the increased categorization of lesions as BI-RADS 3 in women with higher levels of BPE [18]. However, the relationship between MRI experience and the rate of BI-RADS 3 observations has yet to be explored.

Our study focused solely on the diagnostic performance of practicing radiology staff, and we did not evaluate the diagnostic accuracy of trainees, including radiology residents and breast imaging fellows. It is plausible that the greatest amount of improvement occurs during these training stages. A recent survey study conducted in Canada revealed that 26% of residents who had completed at least one breast rotation had not been exposed to breast MRI at all [27]. This finding highlights a potential learning gap early in radiology education that could be addressed to enhance the diagnostic accuracy of breast MRIs.

## 5. Conclusions

Screening MRIs play a crucial role in the early detection of breast cancer in individuals at high risk. It has been observed that greater MRI reading experience enhances the diagnostic accuracy of radiologists when identifying high-risk or malignant lesions, even in cases with increased BPE. To further improve diagnostic accuracy, strategies aimed at enhancing the education of radiology residents and providing continuing education for practicing breast radiologists could be implemented. The negative impact of increased BPE on the diagnostic accuracy of BI-RADS 4/5 lesions is evident, leading to unnecessary biopsies, heightened anxiety, and increased follow-up costs. This highlights the importance of existing measures and the need for future studies to explore additional methods to minimize BPE.

## Figures and Tables

**Table 1:** Summary of high-risk screening MRIs, BI-RADS 4/5 observations biopsied, pathology, and PPV3 stratified by exam year (N = 536).

Exam Year	Total High-Risk Screening MRI Performed	Total BI-RADS 4/5 Observations Biopsied	Malignant	Non-Malignant	PPV3
2011 (1 July to 31 December)	134	8	1	7	0.13
2012	311	31	5	26	0.16
2013	676	51	8	43	0.16
2014	804	71	11	60	0.15
2015	823	73	12	61	0.16
2016	768	70	14	56	0.20
2017	886	71	7	64	0.10
2018	913	57	5	52	0.08
2019	1007	90	8	82	0.09
2020 (1 January– 30 June)	499	14	6	8	0.43
Total	6821	536	77	459	0.14

**Table 2:** Total BI-RADS 4/5 observations from high-risk screening MRI made by individual radiologists (N = 536). * Indicates radiologists with fewer than 10 observations and are excluded for the remainder of the analyses.

Radiologist	Observations	Percent of Total Observations
1	58	10.8%
2 *	6	1.1%
3	106	19.8%
4 *	3	0.6%
5	41	7.6%
6	90	16.8%
7	133	24.8%
8	88	16.4%
9 *	1	0.2%
10 *	5	0.9%
11 *	5	0.9%
Total	536	

**Table 3:** PPV3 stratified by radiologists who made more than 10 observations (N = 516).

Radiologist	Non-Malignant	Malignant	Total	PPV3
1	47	11	58	0.19
3	87	19	106	0.18
5	32	9	41	0.22
6	76	14	90	0.16
7	125	8	133	0.06
8	76	12	88	0.14
Total	443	73	516	0.17

**Table 4:** Single variable logistic regression analysis of variables associated with accuracy (N = 516) * *p* < 0.05.

	Malignant		Malignant or High-Risk Lesions	
	OR	*p*	OR	*p*
**MRI Experience Increase By Different Time Ranges**				
1 year	1.03	0.24	1.05	0.03 *
5 year	1.17	0.24	1.27	0.03 *
10 year	1.31	0.24	1.61	0.03 *
**BPE Levels** (Compared to Marked BPE)				
Minimal BPE	5.6	0.004 *	2.4	0.049 *
Mild BPE	4.2	0.01 *	2.2	0.03 *
Moderate BPE	1.8	0.30	1.3	0.48
**Amount of FGT** (Compared to D—Extremely Dense FGT)				
A—Almost Entirely Fat	4.2	0.008 *	2.8	0.02 *
B—Scattered FGT	3.4	0.004 *	1.9	0.07
C—Heterogeneously Dense FGT	2.1	0.06	2.0	0.02 *

**Table 5:** Multiple variable regression analyses of variables associated with accuracy (N = 516) * *p* < 0.05.

	Malignant		Malignant or High-Risk Lesions	
MODEL 1	OR	*p*	OR	*p*
**MRI Experience** (years)	1.03	0.27	1.05	0.03 *
**BPE Levels** (Compared to Marked BPE)				
Minimal BPE	4.3	0.02 *	2.4	0.08
Mild BPE	3.2	0.04 *	2.2	0.08
Moderate BPE	1.5	0.47	1.3	0.67
**Amount of FGT** (Compared to D—Extremely Dense FGT)				
A—Almost Entirely Fat	2.4	0.11	2.8	0.15
B—Scattered FGT	2.2	0.07	1.9	0.37
C—Heterogeneously Dense FGT	2.0	0.09	2.0	0.03 *
**MODEL 2**				
**BPE Levels** (Compared to Marked BPE)				
Minimal BPE	4.2	0.02 *	2.2	0.10
Mild BPE	3.2	0.04 *	1.9	0.09
Moderate BPE	1.5	0.49	1.1	0.73
**Amount of FGT** (Compared to D—Extremely Dense FGT)				
A—Almost Entirely Fat	2.5	0.10	2.0	0.14
B—Scattered FGT	2.3	0.07	1.4	0.32
C—Heterogeneously Dense FGT	2.0	0.08	1.9	0.03 *
**MODEL 3**				
**MRI Experience** (1 year)	1.03	0.31	1.1	0.04 *
**Amount of FGT** (Compared to D—Extremely Dense FGT)				
A—Almost Entirely Fat	4.1	0.008 *	2.8	0.02 *
B—Scattered FGT	3.3	0.005 *	1.8	0.09
C—Heterogeneously Dense FGT	2.1	0.06	1.9	0.02 *

**Table 6:** Frequencies of levels of BPE and amount of FGT.

BPE	Frequency	Percent
Minimal	51	9.9
Mild	162	31.5
Moderate	233	45.2
Marked	69	13.4
**Amount of FGT**		
A—Almost Entirely Fat	33	6.4
B—Scattered FGT	97	18.8
C—Heterogeneously Dense FGT	259	50.3
D—Extremely Dense FGT	126	24.5

## Data Availability

Data are contained within the article.

## Abbreviations

Background parenchymal enhancement (BPE), fibroglandular tissue (FGT), positive predictive value of biopsies (PPV3), odds ratio (OR), ductal carcinoma in situ (DCIS), lobular carcinoma in situ (LCIS), atypical lobular hyperplasia (ALH), atypical ductal hyperplasia (ADH), flat epithelial atypia (FEA), dynamic contrast enhanced (DCE).
